# Estimating baseline rates of adverse perinatal and neonatal outcomes using a facility-based surveillance approach: A prospective observational study from the WHO Global Vaccine Safety Multi-Country Collaboration on safety in pregnancy

**DOI:** 10.1016/j.eclinm.2022.101506

**Published:** 2022-06-17

**Authors:** Apoorva Sharan, Anke L. Stuurman, Shubhashri Jahagirdar, Varalakshmi Elango, Margarita Riera-Montes, Neeraj Kumar Kashyap, Jorne Biccler, Ramesh Poluru, Narendra Kumar Arora, Mathews Mathai, Punam Mangtani, Hugo Devlieger, Steven Anderson, Barbee Whitaker, Hui-Lee Wong, Allisyn Moran, Christine Guillard Maure, WHO Global Vaccine Safety Multi-Country Collaboration sites

**Affiliations:** a The INCLEN Trust International, New Delhi, India; b Swiss Tropical and Public Health Institute (Swiss TPH), Basel, Switzerland; c University of Basel, Basel, Switzerland; d P95 Pharmacovigilance and Epidemiology, Leuven, Belgium; e Centre for Maternal and Newborn Health, Liverpool School of Tropical Medicine, Liverpool, UK; f Department of Infectious Disease Epidemiology, London School of Hygiene & Tropical Medicine, London, UK; g Universitair Ziekenhuis, Leuven, Belgium; h Center for Biologics Evaluation and Research (CBER), U.S. Food and Drug Administration (FDA), Silver Spring, MD, USA; i Department of Maternal, Newborn, Child and Adolescent Health, World Health Organization, Geneva, Switzerland; j Department of Essential Medicines and Health Products, World Health Organization, Geneva, Switzerland; k WHO Global Vaccine Safety Multi-county Collaboration sites (listed in appendix 1)

**Keywords:** Perinatal outcomes, Neonatal outcomes, Surveillance, Vaccines, Baseline rates, Minimum detectable risk, Pharmacovigilance

## Abstract

**Background:**

Most perinatal and neonatal deaths occur in low- and middle-income countries (LMICs), yet, quality data on burden of adverse outcomes of pregnancy is limited in such countries.

**Methods:**

A network of 21 maternity units, across seven countries, undertook surveillance for low birthweight, preterm birth, small for gestational age (SGA), stillbirths, congenital microcephaly, in-hospital neonatal deaths, and neonatal infections in a cohort of over 85,000 births from May 2019 - August 2020. For each outcome, site-specific rates per 1,000 livebirths (or per 1,000 total births for stillbirth) and 95% confidence intervals (CI) were calculated. Descriptive sensitivity analysis was conducted to gain insight regarding underreporting of four outcomes at 16 sites.

**Findings:**

Estimated rates varied across countries and sites, ranging between 43·3-329·5 and 21·4-276·6/1000 livebirths for low birthweight and preterm birth respectively and 11·8-81/1,000 livebirths for SGA. No cases of congenital microcephaly were reported by three sites while the highest estimated rate was 13/1,000 livebirths. Neonatal infection and neonatal death rates varied between 1·8-73 and 0-59·9/1000 livebirths respectively while stillbirth rates ranged between 0-57·1/1000 total births across study sites. Results from the sensitivity analysis confirmed the underreporting of congenital microcephaly and SGA in our study.

**Interpretation:**

Our study establishes site-specific baseline rates for important adverse perinatal and neonatal outcomes and addresses a critical evidence gap towards improved monitoring of benefits and risks of emerging pregnancy and neonatal interventions.

**Funding:**

The study was sponsored by the World Health Organization with funding from the Bill and Melinda Gates Foundation.

**Research in context**

**Evidence before this study**

We searched for United Nations (agencies) reports or, if not available, systematic review on global, regional and country-specific rates of perinatal and neonatal outcomes in low- and middle-income countries, published before November 2020. Annual estimates of global, regional, and country-specific population-based rates of stillbirth, neonatal sepsis (and other selected neonatal infections) and neonatal deaths were available from the United Nations Inter-agency Group for Child Mortality Estimation and the Global Burden of Disease study groups. Data on the burden of small for gestational age (SGA) and preterm and low birthweight births in LMICs were also available. We could not identify any studies providing global or LIMC specific baseline rates for congenital microcephaly.

**Added value of this study**

In this study, we estimate baseline rates for low birthweight, preterm birth, stillbirth, small for gestational age, congenital microcephaly, in-hospital neonatal death and neonatal infection (including neonatal meningitis, invasive bloodstream and respiratory infection) using a prospective, observational, facility-based surveillance approach across one primary, five secondary and 15 tertiary care hospitals in seven countries, over a 12-month period between May 2019-August 2020. Our study provides high-quality data about the burden of adverse perinatal and neonatal outcomes in LMICs and delivers actionable insights regarding the quality of clinical record keeping in these settings. The study findings informed the development of a publicly available dashboard to estimate minimum detectable risk for selected outcomes over multiple scenarios, thereby strengthening available evidence for future safety evaluations of pregnancy interventions.

**Implications of all the available evidence**

Our findings underscore the need for greater data literacy and inter-sectoral collaboration among healthcare providers, pharmacovigilance and health-programme managers to promote harmonized approaches (case definitions and data elements) for capturing adverse outcomes of pregnancy. The lack of quality data for estimating gestational age affected the reliable identification of five of the seven study outcomes. The use of diverse charts, and cut-off values, and lack protocols for systematic assessment of all births to identify SGA and congenital microcephaly cases contributed to their underreporting, as confirmed by the sensitivity analysis in the study. Despite its limitations, our study establishes the feasibility and suitability of using an active, facility-based surveillance approach for monitoring perinatal and neonatal outcomes in resource-constrained settings in LMICs.

Alt-text: Unlabelled box

## Introduction

An estimated 295,000 maternal and 2·4 million neonatal deaths continue to occur worldwide annually, despite a nearly 40% decline in the past two decades.^1^^,^^2^ An equally significant but often overlooked burden is the nearly two million stillbirths that occur every year.^3^ Beyond mortality, adverse outcomes of pregnancy are associated with significant morbidity and lasting disability; babies born with a low birthweight are at an increased risk of neurodevelopmental impairment, stunting and non-communicable diseases.^4^

The burden of maternal, perinatal, and neonatal morbidity and mortality is not uniform; over 82% of stillbirths and almost all maternal and neonatal deaths occur in low- and middle-income countries (LMICs).^2^^,^^3^ Yet the quality and quantity of information on the burden, underlying causes and determinants of adverse outcomes of pregnancy and early childhood is limited in LMICs.^5^ A majority of neonatal deaths and stillbirths are not registered, civil, and vital registration systems are not well functioning and routine health management information systems may not capture community-based deaths and suffer from data quality issues.^6^^,^^7^ Population-based household surveys such as the Demographic and Health Surveys (DHS) and Multiple Indicator Cluster Surveys (MICS) lack the power to capture morbidity and mortality data at sub-national levels and are susceptible to underreporting and recall bias.^6^^,^^7^ Incomplete or missing clinical documentation, variability in case definitions, outcome identification methods and data elements collected, and the need to track mother-child dyads longitudinally further hamper the generation of reliable and comparable baseline rates for these outcomes.^6^^,^^8^

Most global maternal, perinatal, and neonatal deaths are preventable with increasing equitable access to quality interventions along the continuum of care.^9^ The onset of the COVID-19 pandemic has exacerbated inequities in access to healthcare and disrupted delivery of health services, potentially stalling the progress made in maternal and child survival in the past decades.^10^ Growing evidence supports the use of maternal immunization for reducing maternal, perinatal and neonatal morbidity and mortality.^8^ The World Health Organization's (WHO) immunisation policy for approved COVID-19 vaccines recommends vaccination of pregnant women at high risk of exposure or severe disease from SARS-COV-2.^11^ In the absence of reliable baseline rates, the introduction of newly developed maternal vaccines in countries with limited pharmacovigilance capacity may result in a perceived increase in occurrence of adverse events following immunization. Strengthening the surveillance of adverse outcomes of pregnancy and early childhood using standardized definitions and tools is critical for monitoring progress towards improved maternal and child health targets, evaluating the benefit-risk of emerging pregnancy and neonatal interventions and embedding these interventions at programme level.^12^^,^^13^

The aim of the WHO Global Vaccine Safety Multi-Country Collaboration (GVS MCC) study on safety in pregnancy was to estimate baseline rates and minimum detectable risk of select adverse perinatal and neonatal outcomes using a facility-based international surveillance network across 21 sites in six LMICs (Ghana, United Republic of Tanzania, Zimbabwe, Islamic Republic of Iran, India & Nepal) and one high-income country (Spain). A sub-set of cases of study outcomes were also recruited to assess applicability of standardized case definitions developed by the Global Alignment of Immunization safety Assessment in pregnancy (GAIA) project.^13^ The detailed study results are available in the study report.^14^ In this publication, we present the estimated rates for all study outcomes and results from a sensitivity analysis undertaken to assess the magnitude of underreporting of four out of seven study outcomes. The applicability of the minimum detectable risk^15^ for informing future safety studies is also examined using estimates of low birthweight as an example.

## Methods

### Study design & setting

A prospective observational study design was implemented across 21 sites, including one primary, five secondary and 15 tertiary care hospitals. A multi-step process was implemented for site-selection; 51 potential sites were identified based on previous collaboration or recommendations from Ministries of Health or WHO regional and country offices from LMICs^16^ interested in strengthening their capacity in maternal immunisation safety monitoring. These sites were invited to complete a screening questionnaire evaluating the suitability of their infrastructure, patient record keeping and documentation practices for participating in the study.^16^ Thirty-seven sites were short-listed to participate in a feasibility assessment to evaluate their ability to identify selected neonatal outcomes, document their maternal immunization exposure status and classify them using standardized GAIA case definitions.^16^ For this assessment, sites were asked to complete pre-designed case report forms (CRFs) for at-least one retrospectively identified pre-term birth, neonatal death, neonatal invasive bloodstream infection and stillbirth cases and document their maternal immunization status. Thirty-two sites completed the feasibility assessment and only 26 sites were able to identify at-least one subject per outcome. Out of the 26 sites, two sites withdrew from participation after the assessment, 24 sites were shortlisted for the study and data collection was initiated at 21 sites.^16^ Although the sites underwent a thorough feasibility assessment (described in further detail in a previous publication),^16^ the selection of sites was purposive and not intended to be geographically representative. All but one of the participating sites collected data over 12 consecutive months between May 2019 and August 2020; at one site, data collection was terminated after 8 months due to lack of inter-departmental support and logistical challenges in data collection exacerbated by the COVID-19 pandemic.

### Study outcomes

In consultation with site investigators, seven adverse perinatal and neonatal outcomes were selected based on public health priority and perceived complexity of data collection; low birthweight, preterm birth, small for gestational age (SGA), stillbirth, congenital microcephaly, in-hospital neonatal death and neonatal infection (refer to supplementary material *S1*: definitions used for identification of study outcomes by sites and study statisticians). As specified by the standardized GAIA case definitions, three types of neonatal infections were included for surveillance (meningitis, invasive bloodstream and respiratory infections), based on diagnoses recorded in the patient case records by the treating team, which could be clinical or supported by laboratory testing.^17^

### Outcome identification

All births occurring at participating sites were recorded and screened for identification of study outcomes during the neonatal period, using routinely collected hospital data. All relevant data sources were reviewed; births were ascertained from the birth register in the labour ward while study outcomes were identified by manual review of birth and admission registers as well as patient records from maternity, neonatal or paediatric wards and neonatal intensive care units (NICU). There was no active follow-up to identify outcomes occurring outside the site. However, all babies born at the facility were followed-up in-hospital for outcome identification at subsequent admissions during the neonatal period. Congenital microcephaly and SGA were not ascertained as part of the routine clinical practice at one site (St Francis Regional Hospital, United Republic of Tanzania); therefore, these two outcomes were not studied for this site.

**Figure 1:**
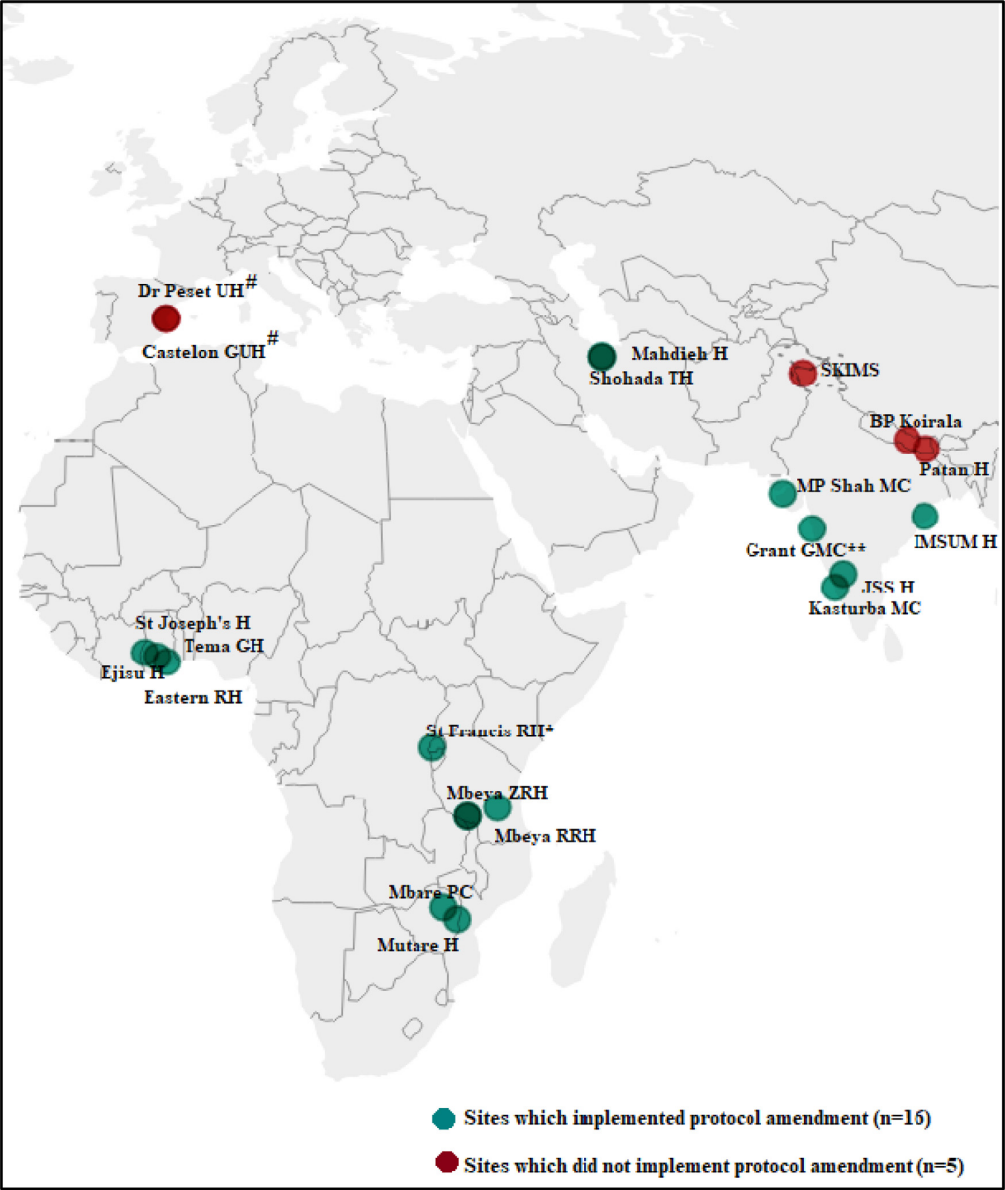
Map of study sites with status of protocol amendment. * St Francis Regional Hospital (United Republic of Tanzania) did not routinely assess births for SGA and congenital microcephaly, hence these two outcomes were not studied for this site. ** Data collection had to be terminated at 8 months at Grant GMC Hospital (India). At all other sites, data was collected for 12 months consecutively. # The registration and screening of individual births was not recorded in the SOMAARTH III application at the two sites in Spain due to lack of approval from EC. This information was submitted in aggregated format by the two sites and recorded in the study application by all other sites. Abbreviations- BP: BP Koirala Institute of Health Sciences; GH: General Hospital; GMC: Government Medical College; GUH: General University Hospital; H: Hospital; IMS SUM: Institute of Medical Science and Sum Hospital; MC: Medical College; PC: Polyclinic; PH: Provincial Hospital; RH: Referral/Regional Hospital; RRH: Regional Referral Hospital; SKIMS: Sher-i-Kashmir Institute of Medical Sciences; TH: Teaching Hospital; UH: University Hospital; ZRH: Zonal Referral Hospital.

Initially, collection of information was restricted to identification of study outcomes during the neonatal period and recording of their date of occurrence by sites. However, through remote data monitoring and on-site visits, it became apparent that sites were underreporting some of the study outcomes. For instance, manual review of the detailed information collected for recruited cases indicated that not all neonates with a birthweight of <2500g or gestational age of <37 weeks were flagged as low birthweight or preterm birth cases respectively. Similarly, comparison of study data against source data during on-site visits helped identify instances of missed identification of study outcomes. To examine the magnitude of underreporting of cases, the protocol was amended to enable the systematic collection of birthweight, gestational age, sex, and head circumference data for all live births. The amendment enabled the study statisticians to identify low birthweight, preterm birth, SGA and congenital microcephaly cases using standardized definitions (refer to supplementary material *S1*: definitions used for identification of study outcomes by sites and study statisticians). Between January and April 2020, 16 of the 21 participating sites obtained approval for the protocol amendment (refer Figure 1 for map of study sites with status of protocol amendments).

For each study outcome, a sub-set of up to 100 cases were recruited in to the study following informed consent from the mother, to assess the applicability of standardized GAIA case definitions. Detailed clinical and maternal immunization exposure information were collected for these recruited cases and the proportion of cases meeting the case definition for study outcomes and maternal immunization were calculated; results of this assessment can be accessed in a separate paper.^18^

### Data collection

A customized Android-based application, SOMAARTH III^19^ was used to collect study data at all but the two participating sites in Spain. The regional Ethics Committee (EC) in Spain did not grant an informed consent waiver for collecting information on all births and study outcomes electronically, therefore the two sites submitted monthly aggregated data of the number of births and study outcomes identified. For all other sites, the application enabled collection of information at birth and during the neonatal period in a linked and anonymized manner. Sites were encouraged to collect data on a daily basis to minimize chances of missing births and study outcomes.

### Data quality assurance & monitoring

A data quality assurance and monitoring plan was developed. The central study team periodically reviewed study data for completeness, quality, and medical congruency and discussed findings with study sites over monthly teleconferences. Data quality assurance visits were performed at 19 of 21 sites. An advisory expert committee maintained scientific oversight during all stages of the study.^20^

### Statistical analysis

For estimating rates and minimum detectable risks, all outcomes identified by the sites during the study period were included. For each outcome, rates per 1,000 livebirths (or per 1,000 total births for stillbirth) were estimated by site. The 95% confidence intervals were calculated using the exact Clopper-Pearson method.^21^

A sensitivity analysis was conducted to assess the impact of the additional information collected per protocol amendment on outcome rates for low birthweight, preterm birth, SGA, and congenital microcephaly. For this analysis, the proportion of cases (along with the 95% CIs) identified by the sites among the total number of cases identified by the sites and statistician was estimated.

The site-specific minimum detectable relative risks for which a two-sided significant test with an alpha of 5% reaches a power of >80% in a cohort study were calculated assuming a 1:3 exposed-unexposed ratio and study duration of two years for stillbirth, preterm birth and low birthweight based on the number of outcomes identified by sites during the 12-month study period. The site-specific minimum detectable odds ratios for which a two-sided significance test with an alpha of 5% reaches a power of >80% in a case-control study were also calculated for the same three study outcomes assuming a case-control ratio of 1:1, study duration of two years and vaccination coverage of 25% among the controls using estimated outcome rates from the study. The 95% confidence intervals were calculated using the exact Clopper-Pearson method.^21^ A dashboard was developed allowing users to explore changes in minimum detectable risk by altering the study duration and either the exposed-unexposed (for cohort studies) or case-control ratio and the vaccination rate (for case-control studies).^22^

Minimum detectable risks were not calculated for the remaining study outcomes due to concerns regarding the internal validity of the number of cases identified by site. All analyses were independently double programmed using R version 3·6·0^23^ by P95 and Stata version 15·1^24^ by the INCLEN Trust International to minimize chances of error and improve the scientific rigour of study results. The double programming outputs were compared and discrepancies were resolved for finalizing study results.

### Ethics

The study protocol and its amendments were approved by the WHO Ethics Review Committee (ERC) and the appropriate local, regional or independent ECs at sites.^14^

All study procedures were conducted according to the International Ethical Guidelines for health-related research involving humans,^22^ under the principles of the Declaration of Helsinki^23^ and considering local legislation on medical research in humans and data sharing from clinical records beyond national or administrative borders. As detailed individual level information was not collected for estimating outcome rates and minimum detectable risk, an informed consent waiver was obtained from the WHO ERC and respective ECs for all but the two sites in Spain, which submitted the number of childbirths and outcomes identified in an aggregated monthly manner.

### Role of funding source

The study has been funded by the Bill & Melinda Gates Foundation. The foundation was not involved in any part of study design, analysis, and interpretation of data, writing, and decision to submit the article for publication. Jorne Biccler and Ramesh Poluru directly accessed and verified the data underlying the results reported in this manuscript. Christine Guillard Maure took the final decision to submit the manuscript for publication.

## Results

During the study period, 85,471 births were recorded and 32,357 study outcomes were identified. There was wide variation in the annual obstetric load at study sites ranging from 864 births at Shohada Teaching Hospital in Islamic Republic of Iran to 10,554 births at BP Koirala Hospital in Nepal.

### Estimated rates

The site characteristics (type of facility and availability of NICU), total number of births and livebirths recorded during the study period and the number and rates of study outcomes per 1000 livebirths (or per 1000 total births for stillbirth) as identified by sites during the study period are presented in Tables 1 and 2, 3 describes the number and proportion of neonatal invasive bloodstream infection, meningitis and respiratory infection among all neonatal infections, as well as their rates per 1000 livebirths, by site.

**Table 1:** Site characteristics, total births, total livebirths, number and rate of stillbirth per 1000 total births and per 1000 livebirths for neonatal death and low birthweight (95% CI) as identified by sites throughout the study period.

Country/Site Name		Type of facility	Total births	Livebirths	Stillbirth		Neonatal Death		Low birthweight	
			N	N	N	Rate (95%CI)	N	Rate (95%CI)	N	Rate (95%CI)
**Ghana**										
St Joseph's H	Secondary		1632	1600	32	19.6 (13.4-27.6)	31	19·4 (13·2-27·4)	220	137·5 (121-155·4)
Ejisu H	Secondary		1441	1426	15	10.4 (5.8-17.1)	0	0 (0-2·6)	89	62·4 (50·4-76·2)
Tema GH	Secondary		5523	5313	210	38 (33.1-43.4)	64	12 (9·3-15·4)	554	104·3 (96·2-112·8)
Eastern RH	Secondary		5386	5238	148	27.5 (23.3-32.2)	103	19·7 (16·1-23·8)	753	143·8 (134·4-153·6)
**United Republic of Tanzania**										
Mbeya ZRH*	Tertiary		7021	6855	166	23.6 (20.2-27.5)	124	18·1 (15·1-21·5)	682	99·5 (92·5-106·8)
St Francis RH*	Tertiary		3484	3373	111	31.9 (26.3-38.2)	71	21 (16·5-26·5)	431	127·8 (116·7-139·5)
Mbeya RRH*	Tertiary		3930	3837	93	23.7 (19.1-28.9)	61	15·9 (12·2-20·4)	321	83·7 (75·1-92·9)
**Zimbabwe**										
Mbare PC*	Primary		5500	5473	27	4.9 (3.2-7.1)	11	2 (1-3·6)	237	43·3 (38·1-49)
Mutare PH*	Tertiary		1558	1469	89	57.1 (46.1-69.8)	88	59·9 (48·3-73·3)	484	329·5 (305·5-354·2)
**Iran (Islamic Republic of)**										
Mahdieh H	Tertiary		5802	5710	92	15.9 (12.8-19.4)	72	12·6 (9·9-15·9)	970	169·9 (160·2-179·9)
Shohada TH	Tertiary		862	836	26	30.2 (19.8-43.9)	16	19·1 (11-30·9)	82	98·1 (78·8-120·3)
**Spain**										
Castellon GUH	Tertiary		1390	1389	1	0.7 (0-4)	3	2·2 (0·4-6·3)	158	113·8 (97·5-131·6)
Dr Peset UH*	Secondary		1078	1078	0	0 (0-3.4)	0	0 (0-3·4)	56	51·9 (39·5-66·9)
**India**										
JSS H	Tertiary		2786	2760	26	9.3 (6.1-13.6)	17	6·2 (3·6-9·8)	487	176·4 (162·4-191·2)
Grant GMC	Tertiary		2247	2151	96	42.7 (34.7-51.9)	78	36·3 (28·8-45·1)	491	228·3 (210·7-246·6)
IMS SUM H	Tertiary		1805	1734	71	39.3 (30.8-49.4)	7	4 (1·6-8·3)	445	256·6 (236·2-277·9)
Kasturba MC	Tertiary		2762	2721	41	14.8 (10.7-20.1)	30	11 (7·5-15·7)	708	260·2 (243·8-277·1)
MP Shah MC	Tertiary		9971	9808	163	16.3 (14-19)	62	6·3 (4·8-8·1)	2421	246·8 (238·3-255·5)
SKIMS	Tertiary		3184	3136	48	15.1 (11.1-19.9)	32	10·2 (7-14·4)	299	95·3 (85·3-106·2)
**Nepal**										
Patan H	Tertiary		7555	7468	87	11.5 (9.2-14.2)	42	5·6 (4·1-7·6)	1064	142·5 (134·6-150·6)
BP Koirala	Tertiary		10554	10333	221	20.9 (18.3-23.9)	28	2·7 (1·8-3·9)	1327	128·4 (122-135)

⁎ Sites with no NICU facility. Abbreviations- BP: BP Koirala of Health Sciences; GH: General Hospital; GMC: Government Medical College; GUH: General University Hospital; H: Hospital; IMS SUM: Institute of Medical Science and Sum Hospital; MC: Medical College; PC: Polyclinic; PH: Provincial Hospital; RH: Referral/Regional Hospital; RRH: Regional Referral Hospital; SKIMS: Sher-i-Kashmir Institute of Medical Sciences; TH: Teaching Hospital; UH: University Hospital; ZRH: Zonal Referral Hospital.

**Table 2:** Total livebirths, number and rate of preterm birth, small for gestational age and congenital microcephaly per 1000 livebirths (95% CI) as identified by sites throughout the study period.

Country/Site Name	Livebirths	Preterm birth		Small for Gestational Age		Congenital Microcephaly		Neonatal Infections	
	N	N	Rate (95%CI)	N	Rate (95%CI)	N	Rate (95%CI)	N	Rate (95%CI)
**Ghana**									
St Joseph's H	1600	107	66·9 (55·1-80·2)	105	65·6 (54-78·9)	2	1·2 (0·2-4·5)	85	53·1 (42·7-65·3)
Ejisu H	1426	45	31·6 (23·1-42)	25	17·5 (11·4-25·8)	2	1·4 (0·2-5·1)	23	16·1 (10·3-24·1)
Tema GH	5313	621	116·9 (108·4-125·8)	341	64·2 (57·7-71·1)	5	0·9 (0·3-2·2)	72	13·6 (10·6-17)
Eastern RH	5238	710	135·5 (126·4-145·1)	247	47·2 (41·6-53·2)	2	0·4 (0-1·4)	52	9·9 (7·4-13)
**United Republic of Tanzania**									
Mbeya ZRH	6855	508	74·1 (68-80·6)	116	16·9 (14-20·3)	1	0·1 (0-0·8)	71	10·4 (8·1-13)
St Francis RH	3373	368	109·1 (98·8-120·1)	NA	NA	NA	NA	74	21·9 (17·3-27·5)
Mbeya RRH	3837	222	57·9 (50·7-65·7)	119	31 (25·8-37)	3	0·8 (0·2-2·3)	97	25·3 (20·5-30·8)
**Zimbabwe**									
Mbare PC	5473	117	21·4 (17·7-25·6)	131	23·9 (20·1-28·3)	0	0 (0-0·7)	10	1·8 (0·9-3·4)
Mutare PH	1469	406	276·4 (253·6-300)	119	81 (67·6-96·2)	0	0 (0-2·5)	7	4·8 (1·9-9·8)
**Iran (Islamic Republic of)**									
Mahdieh H	5710	1177	206·1 (195·7-216·9)	249	43·6 (38·5-49·2)	30	5·3 (3·5-7·5)	64	11·2 (8·6-14·3)
Shohada TH	836	113	135·2 (112·7-160·2)	17	20·3 (11·9-32·4)	8	9·6 (4·1-18·8)	61	73 (56·3-92·7)
**Spain**									
Castellon GUH	1389	137	98·6 (83·5-115·5)	85	61·2 (49·2-75·1)	18	13 (7·7-20·4)	9	6·5 (3-12·3)
Dr Peset UH	1078	56	51·9 (39·5-66·9)	60	55·7 (42·7-71·1)	14	13 (7·1-21·7)	7	6·5 (2·6-13·3)
**India**									
JSS H	2760	386	139·9 (127·1-153·4)	124	44·9 (37·5-53·3)	9	3·3 (1·5-6·2)	62	22·5 (17·3-28·7)
Grant GMC	2151	382	177·6 (161·7-194·4)	45	20·9 (15·3-27·9)	0	0 (0-1·7)	17	7·9 (4·6-12·6)
IMS SUM H	1734	370	213·4 (194·3-233·4)	109	62·9 (51·9-75·3)	6	3·5 (1·3-7·5)	27	15·6 (10·3-22·6)
Kasturba MC	2721	667	245·1 (229·1-261·7)	172	63·2 (54·4-73)	11	4 (2-7·2)	76	27·9 (22·1-34·8)
MP Shah MC	9808	830	84·6 (79·2-90·3)	181	18·5 (15·9-21·3)	9	0·9 (0·4-1·7)	88	9 (7·2-11)
SKIMS	3136	379	120·9 (109·6-132·8)	37	11·8 (8·3-16·2)	1	0·3 (0-1·8)	34	10·8 (7·5-15·1)
**Nepal**									
Patan H	7468	887	118·8 (111·5-126·3)	126	16·9 (14·1-20·1)	3	0·4 (0·1-1·2)	282	37·8 (33·6-42·3)
BP Koirala	10333	580	56·1 (51·8-60·7)	818	79·2 (74-84·5)	15	1·5 (0·8-2·4)	141	13·6 (11·5-16·1)

NA: St Francis RH did not routinely diagnose SGA and congenital microcephaly.

Abbreviations- BP: BP Koirala Institute of Health Sciences; GH: General Hospital; GMC: Government Medical College; GUH: General University Hospital; H: Hospital; IMS SUM: Institute of Medical Science and Sum Hospital; MC: Medical College; NA: Not applicable; PC: Polyclinic; PH: Provincial Hospital; RH: Referral/Regional Hospital; RRH: Regional Referral Hospital; SKIMS: Sher-i-Kashmir Institute of Medical Sciences; TH: Teaching Hospital; UH: University Hospital; ZRH: Zonal Referral Hospital.

**Table 3:** Number of neonatal infections, and number and rates of invasive bloodstream infection, meningitis, and respiratory infection, per 1,000 livebirths, by site.

Country/Site Name	Neonatal Infections	Invasive bloodstream infection		Meningitis		Respiratory infection	
	N	n (% among all NI)	Rate (95% CI)	n (% among all NI)	Rate (95% CI)	n (% among all NI)	Rate (95% CI)
**Ghana**							
St Joseph's H	85	74 (87·1)	46·2 (36·5-57·7)	0 (0)	0 (0-2·3)	11 (12·9)	6·9 (3·4-12·3)
Ejisu H	23	23 (100)	16·1 (10·3-24·1)	0 (0)	0 (0-2·6)	0 (0)	0 (0-2·6)
Tema GH	72	64 (88·9)	12 (9·3-15·4)	2 (2·8)	0·4 (0-1·4)	6 (8·3)	1·1 (0·4-2·5)
Eastern RH	52	49 (94·2)	9·4 (6·9-12·3)	3 (5·8)	0·6 (0·1-1·7)	0 (0)	0 (0-0·7)
**United Republic of Tanzania**							
Mbeya ZRH	71	67 (94·4)	9·8 (7·6-12·4)	2 (2·8)	0·3 (0-1·1)	2 (2·8)	0·3 (0-1·1)
St Francis RH	74	66 (89·2)	19·6 (15·2-24·8)	4 (5·4)	1·2 (0·3-3)	4 (5·4)	1·2 (0·3-3)
Mbeya RRH	97	93 (95·9)	24·2 (19·6-29·6)	2 (2·1)	0·5 (0·1-1·9)	2 (2·1)	0·5 (0·1-1·9)
**Zimbabwe**							
Mbare PC	10	9 (90)	1·6 (0·8-3·1)	0 (0)	0 (0-0·7)	1 (10)	0·2 (0-1)
Mutare PH	7	6 (85·7)	4·1 (1·5-8·9)	0 (0)	0 (0-2·5)	1 (14·3)	0·7 (0-3·8)
**Iran (Islamic Republic of)**							
Mahdieh H	64	52 (81·2)	9·1 (6·8-11·9)	0 (0)	0 (0-0·6)	12 (18·8)	2·1 (1·1-3·7)
Shohada TH	61	53 (86·9)	63·4 (47·8-82·1)	1 (1·6)	1·2 (0-6·6)	7 (11·5)	8·4 (3·4-17·2)
**Spain**							
Castellon GUH	9	7 (77.8)	5 (2-10·4)	1 (11.1)	0·7 (0-4)	1 (11.1)	0·7 (0-4)
Dr Peset UH	7	7 (100)	6·5 (2·6-13·3)	0 (0)	0 (0-3·4)	0 (0)	0 (0)
**India**							
JSS H	62	55 (88·7)	19·9 (15-25·9)	2 (3·2)	0·7 (0·1-2·6)	5 (8·1)	1·8 (0·6-4·2)
Grant GMC	17	14 (82·4)	6·5 (3·6-10·9)	2 (11·8)	0·9 (0·1-3·4)	1 (5·9)	0·5 (0-2·6)
IMS SUM H	27	24 (88·9)	13·8 (8·9-20·5)	3 (11·1)	1·7 (0·4-5)	0 (0)	0 (0-2·1)
Kasturba MC	76	71 (93·4)	26·1 (20·4-32·8)	3 (3·9)	1·1 (0·2-3·2)	2 (2·6)	0·7 (0·1-2·7)
MP Shah MC	88	71 (80·7)	7·2 (5·7-9·1)	1 (1·1)	0·1 (0-0·6)	16 (18·2)	1·6 (0·9-2·6)
SKIMS	34	34 (100)	10·8 (7·5-15·1)	0 (0)	0 (0-1·2)	0 (0)	0 (0-1·2)
**Nepal**							
Patan H	282	112 (39·7)	15 (12·4-18)	51 (18·1)	6·8 (5·1-9)	119 (42·2)	15·9 (13·2-19)
BP Koirala	141	74 (52·5)	7·2 (5·6-9)	24 (17)	2·3 (1·5-3·5)	43 (30·5)	4·2 (3-5·6)

Abbreviations- BP: BP Koirala Institute of Health Sciences; GH: General Hospital; GMC: Government Medical College; GUH: General University Hospital; H: Hospital; IMS SUM: Institute of Medical Science and Sum Hospital; MC: Medical College; PC: Polyclinic; PH: Provincial Hospital; RH: Referral/Regional Hospital; RRH: Regional Referral Hospital; SKIMS: Sher-i-Kashmir Institute of Medical Sciences; TH: Teaching Hospital; UH: University Hospital; ZRH: Zonal Referral Hospital.

The most frequently identified outcomes across sites were low birthweight and preterm birth. The Mbare Polyclinic in Zimbabwe, the only primary care facility in the study, most frequently reported the lowest rates while Mutare Provincial Hospital, also in Zimbabwe, most frequently reported the highest rates for study outcomes.

*Stillbirth*: The rates for stillbirth were the highest at Mutare Provincial Hospital in Zimbabwe (57·1/1000 births).

*Neonatal death*: The rates for neonatal death were also the highest at Mutare Provincial Hospital in Zimbabwe (59·9/1000 livebirths). No neonatal deaths were observed at two sites during the study period (Ejisu Hospital in Ghana and Dr Peset University Hospital in Spain).

*Low birthweight*: Rates for low birthweight were the lowest at Mbare Polyclinic (43·3/1000 livebirths) and highest at Mutare Provincial Hospital (329·5/1,000 livebirths).

*Preterm birth:* Rates for preterm birth were the lowest again at Mbare Polyclinic (21·4/1000 livebirths) and highest at Mutare Provincial Hospital (276·4 /1000 livebirths).

*SGA*: Rates for SGA varied from 11·8/1,000 livebirths at SKIMS, India to 81/1,000 livebirths at Mutare Provincial Hospital, Zimbabwe.

*Congenital microcephaly*: The Mbare Polyclinic and Mutare Provincial Hospital in Zimbabwe, as well as the Grant's Government Medical College in India reported no cases of congenital microcephaly while the highest rates were observed at the two sites in Spain (13/1,000 livebirths at both sites).

*Neonatal infections:* Rates of neonatal infections varied across countries and sites ranging between 1·8/1,000 livebirths at Mbare Polyclinic, Zimbabwe to 73/1000 livebirths at Shohada Teaching Hospital, Islamic Republic of Iran. The most commonly reported type of neonatal infection was invasive bloodstream infection (1·6 -61/1,000 livebirths) followed by respiratory infection (1-15·7/1000 livebirths) and meningitis (0-6·7/1000 livebirths).

### Sensitivity analyses

In Table 4, the total number of outcomes identified (viz. sum of outcomes identified by the sites and study statisticians), the consequent estimated rates per 1000 livebirths, and the number and proportion of outcomes identified by sites alone during the post-protocol amendment period are presented for low birthweight, preterm birth, SGA, and congenital microcephaly cases.

**Table 4:** Total number of outcomes identified (sum of outcomes identified by the sites or study statisticians using post-amendment data; N), and the number, proportion (along with 95% CIs) of outcomes identified by sites alone (n identified by site) for the post-protocol amendment period, by site.

Country/Site Name	Weeks*	Low Birthweight			Preterm birth			Small for gestational age			Congenital microcephaly		
	N	N	Identified by site		N	Identified by site		N	Identified by site		N	Identified by site	
**Ghana**			**N**	**%** **(95% CIs)**		**N**	**%** **(95% CIs)**		**N**	**%** **(95% CIs)**		**N**	**%** **(95% CIs)**
St Joseph's H	18	61	58	95·1% (86·3- 99)	28	28	100% (87·7- 100)	195	29	14·9% (10·2- 20·7)	161	2	1·2% (0·2-4·4)
Ejisu H	18	33	31	93·9% (79·8- 99·3)	14	13	92·9% (66·1- 99·8)	76	11	14·5% (7·5- 24·4)	36	0	0% (0-9·7)
Tema GH	19	200	165	82·5% (76·5- 87·5)	241	219	90·9% (86·5- 94·2)	231	73	31·6% (25·7- 38)	153	1	0·7% (0-3·6)
Eastern RH	19	277	273	98·6% (96·3- 99·6)	280	272	97·1% (94·4- 98·8)	315	87	27·6% (22·8- 32·9)	70	1	1·4% (0-7·7)
**United Republic of Tanzania**													
Mbeya ZRH	16	159	152	95·6% (91·1- 98·2)	226	194	85·8% (80·6- 90·1)	192	49	25·5% (19·5- 32·3)	0	-	-
St Francis RH	16	104	100	96·2% (90·4- 98·9)	123	119	96·7% (91·9- 99·1)	128	0	0% (0-2·8)	0	-	-
Mbeya RRH	16	71	70	98·6% (92·4- 100)	75	49	65·3% (53·5- 76)	74	43	58·1% (46·1- 69·5)	22	0	0% (0- 15·4)
**Zimbabwe**													
Mbare PC	22	78	75	96·2% (89·2- 99·2)	85	44	51·8% (40·7- 62·7)	202	32	15·8% (11·1- 21·6)	9	0	0% (0- 33·6)
Mutare PH	21	172	166	96·5% (92·6- 98·7)	149	138	92·6% (87·2- 96·3)	93	47	50·5% (40- 61·1)	9	0	0% (0- 33·6)
**Iran (Islamic Republic of)**													
Mahdieh H	24	325	321	98·8% (96·9- 99·7)	372	364	97·8% (95·8- 99·1)	151	126	83·4% (76·5- 89)	54	28	51·9% (37·8- 65·7)
Shohada TH	30	31	18	58·1% (39·1- 75·5)	39	36	92·3% (79·1- 98·4)	14	3	21·4% (4·7- 50·8)	9	1	11·1% (0·3- 48·2)
**India**													
JSS H	19	142	137	96·5% (92- 98·8)	128	119	93% (87·1- 96·7)	131	36	27·5% (20- 36)	210	2	1% (0·1-3·4)
Grant GMC	7	120	117	97·5% (92·9- 99·5)	89	88	98·9% (93·9- 100)	122	7	5·7% (2·3- 11·5)	0	-	-
IMS SUM H	17	85	85	100% (95·8- 100)	79	79	100% (95·4- 100)	75	25	33·3% (22·9- 45·2)	2	1	50% (1·3- 98·7)
Kasturba MC	25	264	263	99·6% (97·9- 100)	228	223	97·8% (95- 99·3)	160	65	40·6% (32·9- 48·7)	22	0	0% (0- 15·4)
MP Shah MC	27	923	874	94·7% (93- 96)	387	335	86·6% (82·8- 89·8)	741	55	7·4% (5·6-9·6)	292	3	1% (0·2- 3)

⁎ Duration of study after protocol amendment in weeks. Abbreviations- BP: BP Koirala Institute of Health Sciences; GH: General Hospital; GMC: Government Medical College; H: Hospital; IMS SUM: Institute of Medical Science and Sum Hospital; MC: Medical College; PC: Polyclinic; PH: Provincial Hospital; RH: Referral/Regional Hospital; RRH: Regional Referral Hospital; SKIMS: Sher-i-Kashmir Institute of Medical Sciences; TH: Teaching Hospital; UH: University Hospital; ZRH: Zonal Referral Hospital.

The number of cases identified by the sites never exceeded the number of outcomes identified by study statisticians for any of the outcomes post-amendment. While there was good concurrence between the cases identified by site and study statistician for low birthweight and preterm birth, the majority of cases of SGA and congenital microcephaly were not identified by the sites. Eleven and 14 out of 16 sites identified > 90% of cases of LBW and preterm birth, respectively. The percentage of SGA cases identified by the site ranged from 7·4 to 83·4%, while the percentage of congenital microcephaly cases identified by the site ranged from 0 to 100%.

### Minimum detectable risk

Table 5 describes the site-specific minimum detectable odds ratio for case-control studies and relative risks for cohort studies estimated (along with their 95% CIs), for low birthweight based on the assumptions described in the methods section. The minimum detectable odds ratio and relative risk for low birthweight were below two for 19 of 21 study sites, indicating that for most sites, future vaccine safety studies will be able to detect a less than two-fold increase in low birth weight rates post maternal vaccination at 80% power and 5% two-sided significance over a two-year study period. A dashboard enabling estimation of minimum detectable risks for multiple scenarios has been developed and is accessible at the following link: https://apps.p-95.com/WHO/.^22^

**Table 5:** Estimated minimum detectable odds ratio and relative risk that could be obtained for low birthweight in case-control and cohort studies respectively, along with their 95% CIs, over a two-year study period at 80% power and 5% two-sided significance, by site.

Country/Site Name	Total outcomes identified (N)	Rate per 1000 livebirths	Minimum detectable odds ratio (95% CI)	Minimum detectable relative risk (95% CI)
**Ghana**				
St Joseph's H	220	137·5	1·52 (1·48-1·56)	1·31 (1·29-1·34)
Ejisu H	89	62·4	1·9 (1·8-2·05)	1·55 (1·48-1·63)
Tema H	554	104·3	1·3 (1·29-1·32)	1·19 (1·19-1·2)
Eastern RH	753	143·8	1·26 (1·25-1·27)	1·16 (1·15-1·17)
**United Republic of Tanzania**				
Mbeya ZRH	682	99·5	1·27 (1·26-1·28)	1·17 (1·17-1·18)
St Francis RH	431	127·8	1·35 (1·33-1·37)	1·22 (1·21-1·23)
Mbeya RRH	321	83·7	1·41 (1·39-1·44)	1·26 (1·25-1·28)
**Zimbabwe**				
Mbare PC	237	43·3	1·51 (1·47-1·55)	1·33 (1·3-1·35)
Mutare PH	483	328·8	1·33 (1·31-1·35)	1·17 (1·16-1·19)
**Iran (Islamic Republic of)**				
Mahdieh H	970	169·9	1·22 (1·22-1·23)	1·14 (1·13-1·14)
Shohada TH	82	98·1	1·95 (1·84-2·12)	1·55 (1·48-1·64)
**Spain**				
Castellon GUH	158	113·8	1·63 (1·58-1·7)	2·58 (2·41-2·79)
Dr Peset UH	56	51·9	2·23 (2·05-2·52)	4·56 (3·87-5·51)
**India**				
JSS H	487	176·4	1·33 (1·31-1·34)	1·2 (1·19-1·21)
Grant GMC	491	228·3	1·25 (1·24-1·26)	1·15 (1·14-1·15)
IMS SUM H	445	256·6	1·34 (1·33-1·36)	1·19 (1·18-1·21)
Kasturba MC	708	260·2	1·27 (1·26-1·28)	1·15 (1·15-1·16)
MP Shah MC	2421	246·8	1·14 (1·13-1·14)	1·08 (1·08-1·08)
SKIMS H	299	95·3	1·43 (1·4-1·46)	1·27 (1·25-1·29)
**Nepal**				
Patan H	1064	142·5	1·21 (1·21-1·22)	1·13 (1·13-1·14)
BP Koirala	1327	128·4	1·19 (1·18-1·2)	1·12 (1·12-1·12)

Abbreviations- BP: BP Koirala Institute of Health Sciences; GH: General Hospital; GMC: Government Medical College; GUH: General University Hospital; H: Hospital; IMS SUM: Institute of Medical Science and Sum Hospital; MC: Medical College; PC: Polyclinic; PH: Provincial Hospital; RH: Referral/Regional Hospital; RRH: Regional Referral Hospital; SKIMS: Sher-i-Kashmir Institute of Medical Sciences; TH: Teaching Hospital; UH: University Hospital; ZRH: Zonal Referral Hospital.

## Discussion

**Figure 2:**
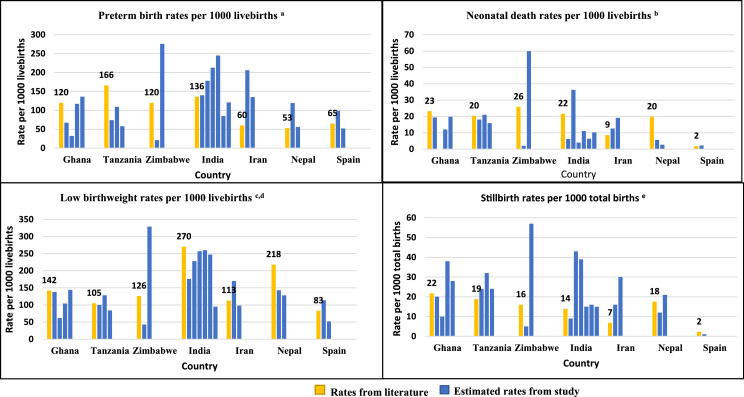
Comparison of preterm birth, low birthweight, neonatal deaths and stillbirth rates from the literature and site-specific rates observed in the study, by country. The yellow bars represent rates from the literature and the blue bars represent rates from study sites, where each bar represents one site. From left to right the sites in **Ghana** are: 1) St Joseph's H; 2) Ejisu H; 3) Tema GH; 4) Eastern RH; **Tanzania**: 1) Mbeya ZRH; 2) St Francis RH; 3) Mbeya RRH; **Zimbabwe**: 1) Mbare PC; 2) Mutare PH; **India**: 1) JSS H; 2) Grant GMC; 3) IMS SUM H; 4) Kasturba MC; 5) MP Shah MC; 6) SKIMS; **Islamic Republic of Iran**: 1) Mahdieh H; 2) Shohada H. **Nepal**: 1) Patan H; 2) BP Koirala; **Spain**: 1) Castellon GUH; 2) Dr Peset UH (*no cases of neonatal death and stillbirth reported by this site*). ^a^Source: Chawanpaiboon S, Vogel JP, Moller A-B, et al. Global, regional, and national estimates of levels of preterm birth in 2014: a systematic review and modelling analysis. The Lancet Global Health 2019; 7(1): e37-e46. ^b^Source: United Nations Inter-agency Group for Child Mortality Estimation (UN IGME). Levels & Trends in Child Mortality: Report. New York, 2020. ^c^Source: United Nations Children's Fund (UNICEF) & World Health Organization (WHO). Low birthweight estimates: levels and trends 2000-2015, 2019. ^d^Low birthweight rates not available for India and Islamic Republic of Iran; hence, rates from South Asia and Middle East/North Africa regions were used for comparison ^e^Source: United Nations Inter-agency Group for Child Mortality Estimation (UN IGME). A neglected tragedy: the global burden of stillbirths 2020.

Our study estimated the site-specific rates for seven important perinatal and neonatal outcomes in 21 geographically diverse sites with varying infrastructure, patient load, and clinical record-keeping practices. National-level estimates are available from WHO and UNICEF for four of the study outcomes; neonatal death,^1^ stillbirth,^3^ preterm birth,^25^ and low birthweight.^26^ Although estimated outcome rates are site-specific and not generalizable at the population level, they fall within the range of available national-level estimates (Figure 2).

There was generally good concurrence between low birthweight and preterm birth cases identified by the sites and study statisticians, reinforcing confidence in the estimated rates for these outcomes. Additional analyses evaluating the applicability of standardized GAIA case definitions resulted in the majority of cases low birthweight, preterm birth and neonatal death cases being classified (to any level of diagnostic certainty) on the basis of case confirmation criteria, suggesting that over-reporting was unlikely for these outcomes.^18^

The identification of study outcomes and subsequent estimation of baseline rates may have been affected by a number of factors, which can be broadly divided into two categories; site- and study-design specific. Site-specific factors include variability in the type of facility and the population they are serving, availability, and quality of site infrastructure and diagnostic capabilities, patient load, methodologies adopted for outcome identification and quality of patient record keeping. The study network comprised mostly tertiary referral hospitals, which are likely to cater to a higher proportion of complicated pregnancies and experience a higher burden of adverse perinatal and neonatal outcomes than the general population. The difference in level of care and availability of technical equipment like electronic weighing scales, ultrasound machines and well-equipped neonatal units significantly affects the type of patients admitted and the ability to accurately assess births for identifying adverse outcomes (refer supplementary material *S2*: availability of technical equipment and study research staff at participating study sites). For instance, the Mutare Provincial Hospital possesses the only well-equipped neonatal unit and serves as a referral centre for seven districts in the Manicaland province in Zimbabwe. The routine practice of primary health care centres such as the Mare Polyclinic to refer mothers with high-risk pregnancies or in preterm labor to the provincial hospital underlies the nearly 10-fold difference in estimated preterm birth rate per 1000 livebirths between the two sites (refer Table 2). The sensitivity analysis and quality assurance visits also highlighted that a greater proportion of neonatal outcomes were missed in the study, particularly at sites with high-patient loads where retrieval of patient data was challenging.

Additional site-specific factors which may have affected outcome identification are, the use of different charts and cut-off values for identifying SGA and congenital microcephaly cases (refer supplementary material *S3*: site reported diagnostic charts used for identifying SGA and congenital microcephaly cases and gestational age cut-offs for viable births) and multiple source documents (e.g. birth registers and patient case records) reporting conflicting information. Finally, some sites did not systematically evaluate all births for study outcomes, for instance, diagnosing SGA cases in routine clinical practice only when the diagnosis was likely to have an impact on patient-case and follow-up protocols.

Important study-specific factors potentially affecting outcome identification included lack of geographical representativeness in site-selection, its observational design and the lack of post-discharge follow-up of births for monitoring study outcomes. The study sites were not selected in a geographically representative manner; which limits the generalizability of the study results. Findings from the sensitivity analyses confirmed that rates of SGA and congenital microcephaly were underestimated in our study. As an observational study, routine clinical practices were not altered at sites and the lack of a uniform approach for case identification may have increased the probability of misclassification and underestimation of the true rate of SGA and congenital microcephaly at sites. The results of the sensitivity analysis were dependent on precise and reliable assessment of data elements (e.g. head circumference), which may have been affected by lack of harmonized procedure across study sites. In our study, surveillance was restricted to study outcomes identified at the study sites. As a result, study outcomes that were not apparent immediately after birth or those that occurred outside the hospital, including neonatal infections and deaths may have been underreported. In comparison to available country-specific or regional estimates, baseline rates of neonatal deaths were lower by a margin of 10% or more in 14 of the 21 sites in our study.^1^ In addition, presenting symptoms for neonatal infections are subtle and their diagnosis may have been affected by variations in standards of care, antibiotic use and availability and quality of diagnostic infrastructure and culture facilities at study sites.^27^^,^^28^

Systematic assessment of gestational age, which affected identification of five of the seven study outcomes, was hindered by a variety of factors, including difficulty in accessing early trimester ultrasound reports at the time of delivery, unreliable quality of ultrasound scans in ANC settings and practice of seeking ANC services post first trimester in communities served by the sites. For the subset of cases recruited to assess applicability of GAIA case definitions, information on gestational age was collected in over 4700 mothers. While ultrasound information from any trimester and LMP date were known for more than half the recruited cases at 17 of the 21 sites, first trimester ultrasound information was only available for more than half the recruited cases at seven sites (refer supplementary material S4: known ultrasound dates and last menstrual period among recruited mothers with completed gestational age information).^18^ Future benefit-risk assessments of pregnancy interventions may benefit from active outreach to obtain poorly documented (e.g. head circumference measurements in routine practice) or difficult to retrieve information (e.g. first or second trimester ultrasound reports for estimation of gestational age).

Lastly, the onset of the COVID-19 pandemic during active data collection in our study temporarily disrupted study activities at many sites and may have affected the number of births and characteristics of mothers attending the site, as well as the risk of adverse outcomes. A recent review of the impact of the COVID-19 pandemic on maternal and perinatal outcomes concluded that the pandemic is associated with a significant increase in maternal deaths and stillbirths.^29^ Although some variations were observed in the number of childbirths occurring at some sites post onset of COVID, these trends were not uniform across sites or over time. Moreover, our study lacked statistical power to detect significant changes between pre- and post- COVID-19 childbirth and outcome rates at sites; any observed changes could not be attributed to the onset of the pandemic alone.

Our study establishes site-specific baseline rates for important neonatal and perinatal outcomes and strengthens available evidence for improved pharmacovigilance of emerging pregnancy and neonatal interventions in LMICs. The estimated minimum detectable risks and dashboard serve as a potentially useful tool to guide future investigators in planning vaccine safety studies. Most large studies estimating population-based rates focus on a subset of neonatal infections (sepsis) and utilize diverse terminologies and case definitions, limiting the comparability of these datasets.^30^^,^^31^ Despite the limitations discussed above, our study estimates site-specific baseline rates for three types of neonatal infections and strengthens the available evidence on neonatal infections in LMIC settings.

Findings from our study also provide valuable insights regarding quality and completeness of routinely collected medical information. The good concurrence between site and study statistician identified low birthweight and preterm birth cases on one hand and evidence of underreporting from sensitivity analysis and on-site visits for selected outcomes such as congenital microcephaly and SGA shed-light on quality of data collected for routine assessment for these outcomes. It underscores the need of stronger coordination mechanisms for standardizing data elements and methods to support harmonized monitoring of maternal interventions across programmes and partners working on improving pregnancy and early childhood health event.

Finally, findings from our study emphasize the need for further training and capacity building of healthcare providers and programme managers across maternal, neonatal and child health (MNCH), immunization and pharmacovigilance programmes as well as health management information systems (HMIS) to promote harmonized approaches for capturing adverse outcomes and using the information for improving the quality of maternal, perinatal and neonatal care. The emergence of novel pregnancy and neonatal interventions, including COVID-19 vaccines, and their potential (or inadvertent) use in pregnant women, reinforces the urgency of improving documentation of gestational age, head circumference, and other elements required to reliably identify adverse outcomes of pregnancy.

## Contributors

**Apoorva Sharan:** Conceptualization, Investigation, Methodology, Visualization, Validation, Writing - original draft, Writing - review & editing. **Anke L Stuurman:** Conceptualization, Investigation, Methodology, Formal analysis, Writing - review & editing. **Shubhashri Jahagirdar:** Investigation, Data curation, Methodology, Formal analysis, Writing - review & editing. **Varalakshmi Elango:** Investigation, Project Administration, Methodology, Writing - review & editing. **Margarita Riera-Montes:** Conceptualization, Investigation, Supervision, Resources, Methodology, Writing - review & editing. **Neeraj Kumar Kashyap**: Software, Data curation, Writing - review & editing. **Jorne Biccler**: Data curation, Formal analysis, Visualization, Validation, Writing- review & editing. **Ramesh Poluru**: Formal analysis, Data curation, Validation, Writing - review & editing. **Jorne Biccler** and **Ramesh Poluru** directly accessed and verified the data underlying the results reported in this manuscript. **Narendra Kumar Arora**: Conceptualization, Methodology, Resources, Writing - review & editing. **Mathews Mathai**: Conceptualization, Methodology, Writing - review & editing. **Punam Mangtani**: Conceptualization, Methodology, Writing - review & editing. **Hugo DeVlieger**: Conceptualization, Methodology, Writing - review & editing. **Steven Anderson**: Conceptualization, Methodology, Writing - review & editing. **Barbee Whitaker**: Conceptualization, Methodology, Writing - review & editing. **Hui-Lee Wong**: Methodology, Writing - review & editing. **Allysin Moran**: Visualization, Writing - review & editing. **Christine Guillard Maure**: Conceptualization, Methodology, Funding acquisition, Project administration, Supervision, Writing- review and editing. **Christine Guillard Maure** took the final decision to submit the manuscript for publication. **WHO Global Vaccine Safety Multi-Country Collaboration sites**: Conceptualization, Investigation, Writing- review and editing.

## Data sharing statement

The site-specific and national level cleaned datasets, including the de-identified individual participant data and data dictionary that underlie the results reported in this article have been shared with site principal investigators and national focal points for further analysis and publications in accordance with the data management plan. Researchers who provide a methodologically sound proposal for secondary analyses may approach the corresponding author for access to de-identified data at any time beginning 12 months following the publication of this article. Additional study documents, including the study protocol and the statistical analysis plan can be made available to researchers upon request to the corresponding author.

## Declaration of interests

Margarita Riera-Montes (P95) & Christine Guillard Maure (WHO) declare that their institutions received funding support for this manuscript from the WHO and the Bill & Melinda Gates Foundation respectively. All the remaining authors declare that they have no known competing financial interests or personal relationships that could have appeared to influence the work reported in this paper.

## Appendix Supplementary materials

Image, application 1

Image, application 2

## References

[1] United Nations Inter-agency Group for Child Mortality Estimation (UN IGME) (2020).

[2] World Health Organization (WHO) (2019).

[3] United Nations Inter-agency Group for Child Mortality Estimation (UN IGME). A neglected tragedy: the global burden of stillbirths 2020.

[4] Lawn JE, Blencowe H, Oza S (2014). Every newborn: progress, priorities, and potential beyond survival. Lancet North Am Ed.

[5] World Health Organization (2014).

[6] Lackritz EM SM, Stergachis A (2021).

[7] Kasasa S, Natukwatsa D, Galiwango E (2021). Birth, stillbirth and death registration data completeness, quality and utility in population-based surveys: EN-INDEPTH study. Popul Health Metrics.

[8] Omer SB. (2017). Maternal immunization. N Engl J Med.

[9] Bhutta ZA, Das JK, Bahl R (2014). Can available interventions end preventable deaths in mothers, newborn babies, and stillbirths, and at what cost?. Lancet North Am Ed.

[10] Roberton T, Carter ED, Chou VB (2020). Early estimates of the indirect effects of the COVID-19 pandemic on maternal and child mortality in low-income and middle-income countries: a modelling study. Lancet Glob Health.

[11] World Health Organization (WHO) Stategic Advisory Group of Experts (SAGE) Working Group on COVID-19 Vaccines. Background paper on Covid-19 disease and vaccines, 2020.

[12] Zuber PLF, Moran AC, Chou D (2018). Mapping the landscape of global programmes to evaluate health interventions in pregnancy: the need for harmonised approaches. Stand Tools.

[13] Bonhoeffer J, Kochhar S, Hirschfeld S (2016). Global alignment of immunization safety assessment in pregnancy – the GAIA project. Vaccine.

[14] Stuurman A, Elango V, Riera M, et al. Global Vaccine Safety Multi Country collaboration project measuring risks of early childhood morbid conditions and assessing standardized methods. 2021.https://apps.p-95.com/WHO/STUDY_INFO/WHO%20GVS%20MCC%20Study%20report%20with%20annex%20v1.0.pdf. Accessed 1 February 2022.

[15] Woodward M. (1992). Formulae for sample size, power and minimum detectable relative risk in medical studies. J R Stat Soc Ser D (The Statistician).

[16] Stuurman AL, Riera M, Lamprianou S (2018). Vaccine safety surveillance in pregnancy in low- and middle-income countries using GAIA case definitions: a feasibility assessment. Vaccine.

[17] Vergnano S, Buttery J, Cailes B (2016). Neonatal infections: case definition and guidelines for data collection, analysis, and presentation of immunisation safety data. Vaccine.

[18] Stuurman AL, Sharan A, Jahagirdar S (2021). WHO global vaccine safety multi-country collaboration project on safety in pregnancy: assessing the level of diagnostic certainty using standardized case definitions for perinatal and neonatal outcomes and maternal immunization. Vaccine: X.

[19] INCLEN (2021). http://inclentrust.org/inclen/somaarth-3/.

[20] Sharan A, Jahagirdar S, Stuurman AL, et al. Operational lessons learned in conducting an international study on pharmacovigilance in pregnancy in resource-constrained settings: the WHO global vaccine safety multi-country collaboration project. *Vaccine: X*2022:100160. 10.1016/j.jvacx.2022.100160 PMC8993756 35434599

[21] Clopper CJ, and Pearson ES. The use of confidence or fiducial limits illustrated in the case of the Binomial. *Biometrika*, 1934;26(4):404–413. *JSTOR*, 10.2307/2331986. Accessed 5 June 2022.

[22] WHO GVS MCC Dashboard – minimum detectable risk. 2021. https://apps.p-95.com/WHO/. Accessed 1 July 2021.

[23] R-3.6.0 for Windows. The R-project for statistical computing. 2021. https://cran.r-project.org/bin/windows/base/old/3.6.0/. Accessed 1 July 2021

[24] StataCorp (2017).

[25] Chawanpaiboon S, Vogel JP, Moller A-B (2019). Global, regional, and national estimates of levels of preterm birth in 2014: a systematic review and modelling analysis. Lancet Glob Health.

[26] United Nations Children's Fund (UNICEF) & World Health Organization (WHO). Low birthweight estimates: levels and trends 2000-2015, 2019.

[27] Thaver D, Zaidi AK. (2009). Burden of neonatal infections in developing countries: a review of evidence from community-based studies. Pediatr Infect Dis J.

[28] Zea-Vera A, Ochoa TJ. (2015). Challenges in the diagnosis and management of neonatal sepsis. J Trop Pediatr.

[29] Chmielewska B, Barratt I, Townsend R, et al. Effects of the COVID-19 pandemic on maternal and perinatal outcomes: a systematicreview and meta-analysis. [published correction appears in Lancet Glob Health. 2021;9(6):e758]. *Lancet Glob Health*. 2021;9(6):e759–e772. 10.1016/S2214-109X(21)00079-6 PMC8012052 33811827

[30] Liu L, Oza S, Hogan D (2016). Global, regional, and national causes of under-5 mortality in 2000–15: an updated systematic analysis with implications for the Sustainable Development Goals. Lancet North Am Ed.

[31] Fleischmann C, Reichert F, Cassini A (2021). Global incidence and mortality of neonatal sepsis: a systematic review and meta-analysis. Arch Dis Child.

