# Supplementary material for: Estimating baseline rates of adverse perinatal and neonatal outcomes using a facility-based surveillance approach: A prospective observational study from the WHO Global Vaccine Safety Multi-Country Collaboration on safety in pregnancy
Source: eClinicalMedicine. 2022 Jun 17;50:101506. doi: 10.1016/j.eclinm.2022.101506 (PMC9234094; doi:10.1016/j.eclinm.2022.101506)
Supplement: Supplementary file 1 [file mmc1.docx]

**WHO Global Vaccine Safety Multi-Country Collaboration Sites**

| **Sl no.** | **Country** | **First name** | **Middle name(s)** | **Last name** | **Affiliation** |
| --- | --- | --- | --- | --- | --- |
| 1 | Ghana | Joseph | Horatius Kojo | Donkor | Tema General Hospital, Tema, Ghana |
| 2 | Ghana | Richard | - | Wodah-Seme | St. Joseph’s Hospital, Jirapa, Ghana |
| 3 | Ghana | Kwasi | Baffour | Gyimah | Ghana Health Service: Ejisu Government Hospital, Ejisu, Ghana |
| 4 | Ghana | Seth | - | Twum | Pharmacy department, Eastern Regional Hospital, Ghana |
| 5 | United Republic of Tanzania | Issa | - | Sabi | National Institute for Medical Research (NIMR), Mbeya Medical Research Center, Mbeya, United Republic of Tanzania |
| 6 | United Republic of Tanzania | Rebecca | - | Mokeha | Mbeya Zonal Referral Hospital, Mbeya, United Republic of Tanzania |
| 7 | United Republic of Tanzania | Elias | - | Kweyamba | St. Francis Referral Hospital, Ifakara, United Republic of Tanzania |
| 8 | United Republic of Tanzania | Henry | - | Marique | St. Francis Referral Hospital, Ifakara, United Republic of Tanzania |
| 9 | United Republic of Tanzania | Ismail | - | Macha | Mbeya Regional Referral Hospital, Mbeya, United Republic of Tanzania |
| 10 | Zimbabwe | Jaensch | Masanga | Mutede | Mutare Provincial Hospital, Ministry of Health and Child Care, Zimbabwe |
| 11 | Zimbabwe | Prosper | - | Chonzi | City of Harare, Health Department, Ministry of Health and Child Care (for Mbare Provincial Hospital), Zimbabwe |
| 12 | Islamic Republic of Iran | Maryam | - | Shariati | Clinical Research Development Center, Mahdiyeh Educational hospital, Shahid Beheshti University of Medical Sciences, Tehran, Islamic Republic of Iran |
| 13 | Islamic Republic of Iran | Elahe | Rastkar | Mehrabani | Clinical Research Development Center, Mahdiyeh Educational hospital, Shahid Beheshti University of Medical Sciences, Tehran, Islamic Republic of Iran |
| 14 | Spain | Alejandro | - | Orrico-Sánchez | Vaccine Research Department, Fundación para el Fomento de la Investigación Sanitaria y Biomédica de la Comunitat Valenciana, FISABIO-Public Health, Valencia, Spain |
| 15 | Spain | Antonio | - | Carmona | Vaccine Research Department, Fundación para el Fomento de la Investigación Sanitaria y Biomédica de la Comunitat Valenciana, FISABIO-Public Health, Valencia, Spain |
| 16 | Spain | Dafina | - | Petrova | Vaccine Research Department, Fundación para el Fomento de la Investigación Sanitaria y Biomédica de la Comunitat Valenciana, FISABIO-Public Health, Valencia, Spain |
| 17 | India | Leslie | - | Lewis | Kasturba Medical College, Manipal Academy of Higher Education, Manipal, India |
| 18 | India | Muralidhar | - | Pai | Kasturba Medical College, Manipal Academy of Higher Education, Manipal, India |
| 19 | India | Shyamla | - | G | Kasturba Medical College, Manipal Academy of Higher Education, Manipal, India |
| 20 | India | Jyothi | - | Shetty | Kasturba Medical College, Manipal Academy of Higher Education, Manipal, India |
| 21 | India | Akhila | - | Hebbar | Kasturba Medical College, Manipal Academy of Higher Education, Manipal, India |
| 22 | India | Sripad | - | Hebbar | Kasturba Medical College, Manipal Academy of Higher Education, Manipal, India |
| 23 | India | Prathap | - | Kumar | Kasturba Medical College, Manipal Academy of Higher Education, Manipal, India |
| 24 | India | Bhadresh | R | Vyas | MP Shah Government Medical College, Jamnagar, India |
| 25 | India | Lalit | - | Sankhe | Grant Medical College, Mumbai, India |
| 26 | India | Rachita | - | Sarangi | Indian Institute of Medical Sciences and SUM hospital, Bhubaneswar, Orissa, India |
| 27 | India | Jagdish | Prasad | Sahoo | Indian Institute of Medical Sciences and SUM hospital, Bhubaneswar, Orissa, India |
| 28 | India | Mandyam | D | Ravi | JSS Academy of Higher Education and Research, Mysuru, Karnataka, India |
| 29 | India | H V | - | Prajwala | JSS Academy of Higher Education and Research, Mysuru, Karnataka, India |
| 30 | India | Javeed | Iqbal | Bhat | Sher-i-Kashmir Institute of Medical Sciences Srinagar Jammu & Kashmir, India |
| 31 | India | Bashir | Ahmad | Charoo | Sher-i-Kashmir Institute of Medical Sciences Srinagar Jammu & Kashmir, India |
| 32 | Nepal | Nisha | Keshary | Bhatta | B.P.Koirala Institute of Health Sciences, Dharan, Nepal |
| 33 | Nepal | Shyam | Prasad | Kafle | B.P.Koirala Institute of Health Sciences, Dharan, Nepal |
| 34 | Nepal | Mukesh | - | Bhatta | B.P.Koirala Institute of Health Sciences, Dharan, Nepal |
| 35 | Nepal | Mohan | Chandra | Regmi | B.P.Koirala Institute of Health Sciences, Dharan, Nepal |
| 36 | Nepal | Prerana | - | Kansakar | Patan Academy of Health Sciences, Nepal |
| 37 | Nepal | Ganesh | - | Shah | Patan Academy of Health Sciences, Nepal |
