# Supplementary material for: Estimating baseline rates of adverse perinatal and neonatal outcomes using a facility-based surveillance approach: A prospective observational study from the WHO Global Vaccine Safety Multi-Country Collaboration on safety in pregnancy
Source: eClinicalMedicine. 2022 Jun 17;50:101506. doi: 10.1016/j.eclinm.2022.101506 (PMC9234094; doi:10.1016/j.eclinm.2022.101506)
Supplement: Supplementary file 2 [file mmc2.docx]

**Supplementary tables**

**Supplementary table 1 (S1): Definitions used for identification of study outcomes**

| Study outcomes | Definitions used by sites | Definitions used by study statisticians |
| --- | --- | --- |
| Congenital microcephaly^1^* | Postnatal diagnosis of congenital microcephaly (livebirths only) recorded in the patient records | Head circumference <3^rd^ percentile based on the Intergrowth-21 head circumference standards by gestational age for boys and for girls ^2^** |
| Low birthweight^3^ | First weight of <2,500g recorded in the birth register or patient record | Birthweight reported as <2,500g |
| Preterm birth^4^ | Reported gestational age (GA) of <37 weeks | GA reported as <37 weeks |
| Neonatal deaths^5^ | In-hospital death of a live born child within 28 days of birth recorded in the patient record | Not applicable |
| Neonatal infections^6^ | Diagnosis of an invasive bloodstream infection, respiratory infection or meningitis within 28 days of birth, as recorded in the patient records, which could be clinical or supported by laboratory testing. | Not applicable |
| Small for gestational age^7^* | Diagnosis of small for gestational age or small for date recorded in the patient records | Birthweight < 10^th^ percentile based on Intergrowth-21 birthweight standards by gestational age for boys and for girls^8^** |
| Stillbirth^9^* | Fetal death (ante- or intrapartum) occurring before birth after a selected site-specified duration of gestation as recorded in the patient records. | - |

* The diagnostic charts used by sites for identifying congenital microcephaly and SGA cases, as well as the gestational age cut-off used for stillbirths are specified in supplementary material **S3.**

**Selection of charts based on GAIA case definitions. In case of ambiguous sex or missing information on sex, the Intergrowth-21 head circumference standards for boys were used (to minimize chances of missing outcomes)

**Supplementary table 2 (S2): Availability of technical equipment and study research staff at participating study sites**

|  | Availability of technical equipment | | | | Source of records | N study research staff |
| --- | --- | --- | --- | --- | --- | --- |
| Country/  Site Name | **Laboratory** | **Ultrasound** | **X-ray** | **Electronic scale** | **Electronic or paper or combination** |  |
| Ghana |  |  |  |  |  |  |
| St Joseph's H | Yes | Yes | Yes | No | Paper | 6 |
| Ejisu H | Yes | Yes | Yes | Yes | Paper | 6 |
| Tema GH | Yes | Yes | Yes | Yes | Paper | 2 |
| Eastern RH | Yes | Yes | Yes | Yes | Combination | 7 |
| United Republic of Tanzania | |  |  |  |  |  |
| Mbeya ZRH | Yes | Yes | Yes | No | Combination | 4 |
| St Francis RH | Yes | Yes | Yes | No | Paper | 4 |
| Mbeya RRH | Yes | Yes | Yes | Yes | Paper | 3 |
| Zimbabwe |  |  |  |  |  |  |
| Mbare PC | Yes | No | No | No | Paper | 4 |
| Mutare PH | Yes | Yes | Yes | Yes | Paper | >10 |
| Iran (Islamic Republic of) | | | | |  |  |
| Shohada TH | Yes | Yes | Yes | Yes | Combination | 3 |
| Mahdieh H | Yes | Yes | Yes | Yes | Combination | 2 |
| Spain |  |  |  |  |  |  |
| Castellon GUH | Yes | Yes | Yes | Yes | Electronic | 5 |
| Dr Peset UH | Yes | Yes | Yes | Yes | Electronic | 3 |
| India |  |  |  |  |  |  |
| JSS H | Yes | Yes | Yes | Yes | Combination | 3 |
| Grant GMC | Yes | Yes | Yes | Yes | Paper | 4 |
| IMS SUM H | Yes | Yes | Yes | Yes | Combination | 2 |
| Kasturba MC | Yes | Yes | Yes | Yes | Combination | 8 |
| SKIMS | Yes | Yes | Yes | Yes | Paper | 3 |
| MP Shah MC | Yes | Yes | Yes | Yes | Paper | 6 |
| Nepal |  |  |  |  |  |  |
| Patan H | Yes | Yes | Yes | Yes | Paper | 1 |
| BP Koirala | Yes | Yes | Yes | Yes | Combination | 3 |

**Supplementary table 3 (S3): Diagnostic charts used for small for gestational age and congenital microcephaly diagnosis, and gestational age threshold for viable birth, as reported by sites**

| Country/  Site Name | What chart (if any) is used by staff to diagnose a baby being small for gestational age? | What chart (if any) is used by staff to diagnose congenital microcephaly, postnatally? | What is the number of completed weeks of gestation at which you would classify birth as viable? |
| --- | --- | --- | --- |
| Ghana |  |  |  |
| St Joseph's H | WHO SGA chart | WHO head circumference for age chart | 28 |
| Ejisu H | None | None | 28 |
| Tema GH | Using the case definition chart. | Using the case definition chart | 28 |
| Eastern RH | None | WHO Head- circumference-for age chart | 28 |
| United Republic of Tanzania | |  |  |
| Mbeya ZRH | Fenton growth chart | Fenton growth chart | 28 |
| St Francis RH | Weight for age | Head circumference | 28 |
| Mbeya RRH | None | None | 28 |
| Zimbabwe |  |  |  |
| Mbare PC | Graph in the maternity booklet | None | 28 |
| Mutare PH | There is a chart on newborn examination, which was used. Definition of small for gestational age was less that 3rd centile in that chart. | None | 28 |
| Iran (Islamic Republic of) | | | |
| Shohada TH | Lubchenco's charts and accessory WHO's charts | Lubchenco's charts and accessory WHO's chart | 26 |
| Mahdieh H | WHO growth chart (boy and girl) | WHO growth chart (boy and girl) | 25 |
| Spain |  |  |  |
| Castellon GUH | WHO charts | WHO charts | 25 |
| Dr Peset UH | WHO charts | WHO charts | 25 |
| India |  |  |  |
| JSS H | Fenton’s growth chart | Fenton’s growth chart | 27 |
| Grant GMC | None | None | 28 |
| IMS SUM H | Fenton Intrauterine Growth Chart | Fenton Intrauterine Growth Chart | 25 |
| Kasturba MC | WHO/Lubchenco | WHO/Lubchenco | 24 |
| SKIMS | Fenton | Fenton | 28 |
| MP Shah MC | Modified Ballard chart | Head circumference manually | 22 |
| Nepal |  |  |  |
| Patan H | Baby's growth charts and charts | Baby's growth charts and charts | 25 |
| BP Koirala | Intergrowth & WHO | Intergrowth | 22 |

Abbreviations- BP: BP Koirala Institute of Health Sciences; GH: General Hospital; GMC: Government Medical College; GUH: General University Hospital; H: Hospital; IMS SUM: Institute of Medical Science and Sum Hospital; MC: Medical College; PC: Polyclinic; PH: Provincial Hospital; RH: Referral/Regional Hospital; RRH: Regional Referral Hospital; SKIMS: Sher-i-Kashmir Institute of Medical Sciences; TH: Teaching Hospital; UH: University Hospital; ZRH: Zonal Referral Hospital

**Supplementary table 4 (S4): Availability of information on ultrasound and LMP dates among recruited mothers with gestational age information collected**

| **Site name** | **Mothers, N** | **1^st^ trimester ultrasound n (%)** | **1^st^ or 2^nd^ trimester ultrasound**  **n (%)** | **1^st^, 2^nd^ or 3^rd^ trimester ultrasound**  **n (%)** | **LMP date known**  **n (%)** |
| --- | --- | --- | --- | --- | --- |
| **Ghana** |  |  |  |  |  |
| St Joseph's H | 198 | 0 (0) | 165 (83·3) | 189 (95·4) | 43 (21·7) |
| Ejisu H | 57 | 27 (47·3) | 47 (82·4) | 55 (96·4) | 33 (57·8) |
| Tema GH | 336 | 8 (2·3) | 31 (9·2) | 48 (14·3) | 5 (1·5) |
| Eastern RH | 365 | 126 (34·5) | 274 (75) | 328 (89·8) | 63 (17·2) |
| **United Republic of Tanzania** | | |  |  |  |
| Mbeya ZRH | 325 | 12 (3·6) | 46 (14·1) | 187 (57·5) | 273 (84) |
| St Francis RH | 240 | 0 (0) | 4 (1·6) | 55 (23) | 188 (78·3) |
| Mbeya RRH | 300 | 2 (0·6) | 11 (3·6) | 115 (38·3) | 271 (90·3) |
| **Zimbabwe** |  |  |  |  |  |
| Mbare PC | 159 | 0 (0) | 8 (5) | 9 (5·6) | 151 (94·9) |
| Mutare PH | 241 | 5 (2·1) | 46 (19) | 118 (49) | 197 (82) |
| **India** |  |  |  |  |  |
| JSS H | 197 | 113 (57·3) | 141 (71·5) | 171 (86·8) | 191 (96·9) |
| Grant GMC | 175 | 26 (14·8) | 104 (59·4) | 161 (92) | 137 (78·2) |
| IMS SUM H | 231 | 42 (18·2) | 124 (53·6) | 229 (99·1) | 227 (98·2) |
| Kasturba MC | 240 | 164 (68·3) | 192 (80) | 235 (97·9) | 237 (98·7) |
| MP Shah MC | 309 | 37 (11·9) | 121 (39·1) | 181 (58·5) | 238 (77) |
| SKIMS | 197 | 54 (27·4) | 165 (83·7) | 195 (98·9) | 195 (98·9) |
| **Iran (Islamic Republic of)** | | | | | |
| Mahdieh H | 349 | 239 (68·4) | 304 (87·1) | 321 (91·9) | 249 (71·3) |
| Shohada TH | 131 | 90 (68·7) | 98 (74·8) | 102 (77·8) | 15 (11·4) |
| **Nepal** |  |  |  |  |  |
| Patan H | 196 | 120 (61·2) | 179 (91·3) | 189 (96·4) | 191 (97·4) |
| BP Koirala | 320 | 110 (34·3) | 218 (68·1) | 292 (91·2) | 306 (95·6) |
| **Spain** |  |  |  |  |  |
| Castellon GUH | 138 | 111 (80·4) | 116 (84) | 122 (88·4) | 130 (94·2) |
| Dr Peset UH | 76 | 56 (73·6) | 63 (82·8) | 68 (89·4) | 71 (93·4) |

Abbreviations- LMP: Last menstrual period; BP: BP Koirala Institute of Health Sciences; GH: General Hospital; GMC: Government Medical College; GUH: General University Hospital; H: Hospital; IMS SUM: Institute of Medical Science and Sum Hospital; MC: Medical College; PC: Polyclinic; PH: Provincial Hospital; RH: Referral/Regional Hospital; RRH: Regional Referral Hospital; SKIMS: Sher-i-Kashmir Institute of Medical Sciences; TH: Teaching Hospital; UH: University Hospital; ZRH: Zonal Referral Hospital

References

1. DeSilva M, Munoz FM, Sell E, et al. Congenital microcephaly: Case definition & guidelines for data collection, analysis, and presentation of safety data after maternal immunisation. *Vaccine* 2017; **35**(48 Pt A): 6472-82.

2. INTERGROWTH-21st. Birthweight standards. Available at: <http://intergrowth21.ndog.ox.ac.uk/>, (Accessed 21.07.2021).

3. Cutland CL, Lackritz EM, Mallett-Moore T, et al. Low birth weight: Case definition & guidelines for data collection, analysis, and presentation of maternal immunization safety data. *Vaccine* 2017; **35**(48 Pt A): 6492-500.

4. Quinn J-A, Munoz FM, Gonik B, et al. Preterm birth: Case definition & guidelines for data collection, analysis, and presentation of immunisation safety data. *Vaccine* 2016; **34**(49): 6047-56.

5. Pathirana J, Munoz FM, Abbing-Karahagopian V, et al. Neonatal death: Case definition & guidelines for data collection, analysis, and presentation of immunization safety data. *Vaccine* 2016; **34**(49): 6027-37.

6. Vergnano S, Buttery J, Cailes B, et al. Neonatal infections: Case definition and guidelines for data collection, analysis, and presentation of immunisation safety data. *Vaccine* 2016; **34**(49): 6038-46.

7. Schlaudecker EP, Munoz FM, Bardají A, et al. Small for gestational age: Case definition & guidelines for data collection, analysis, and presentation of maternal immunisation safety data. *Vaccine* 2017; **35**(48 Pt A): 6518-28.

8. INTERGROWTH-21st. Head circumference standards. Available at: <http://intergrowth21.ndog.ox.ac.uk/>, (Accessed 21.07.2021).

9. Tavares Da Silva F, Gonik B, McMillan M, et al. Stillbirth: Case definition and guidelines for data collection, analysis, and presentation of maternal immunization safety data. *Vaccine* 2016; **34**(49): 6057-68.
